# Supplementary material for: Optimizing 5’UTRs for mRNA-delivered gene editing using deep learning
Source: Nat Commun. 2024 Jun 20;15:5284. doi: 10.1038/s41467-024-49508-2 (PMC11189900; doi:10.1038/s41467-024-49508-2)
Supplement: Supplementary file 1 — Supplementary Information [file 41467_2024_49508_MOESM1_ESM.pdf]

Supplementary Information for

Optimizing 5'UTRs for mRNA-delivered gene editing using deep learning

Sebastian Castillo Hair, Stephen Fedak, Ban Wang, Johannes Linder, Kyle Havens, Michael Certo, Georg Seelig

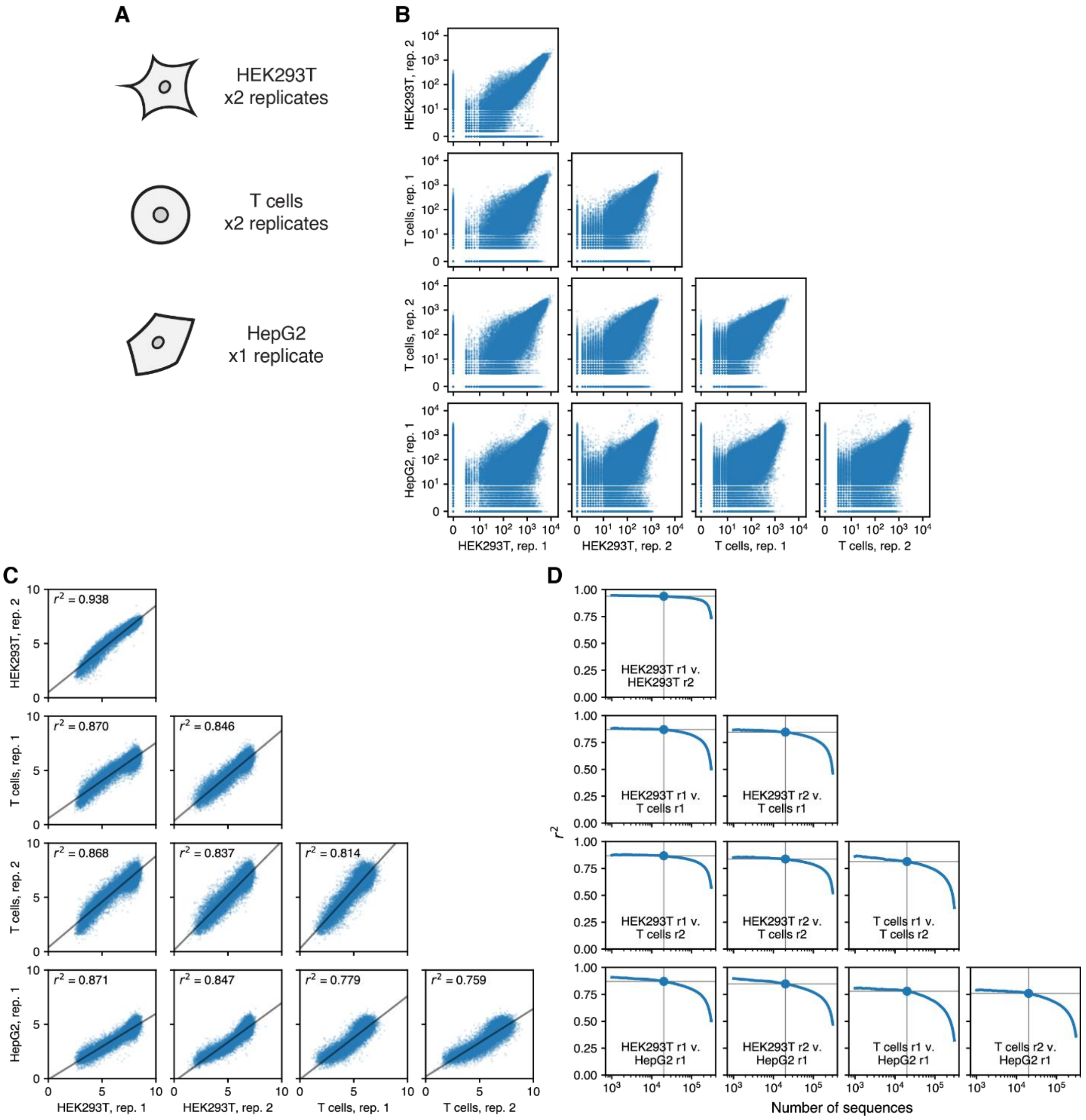

Supplementary Figure 1. Comparison of polysome profiling MPRA data in HEK293, T cells, and HepG2. (A) Cell lines and number of biological replicates. **(B)** Comparison of number of reads for each 5'UTR sequence across all pairs of replicates. **(C)** MRL comparison for the 20,000 5'UTR sequences with the highest minimum read coverage across all replicates. A regression line for each pair is shown in black along with the coefficient of determination r^2. **(D)** r^2 as a function of the number of sequences used. Sequences were sorted by the minimum number of reads across all replicates in descending order. Then, the top x sequences (x axis) were used to calculate a corresponding r^2 value (y axis). The large marker and the gray lines indicate the number of sequences and r^2 in **(C)**. Source data are provided as a Source Data file.

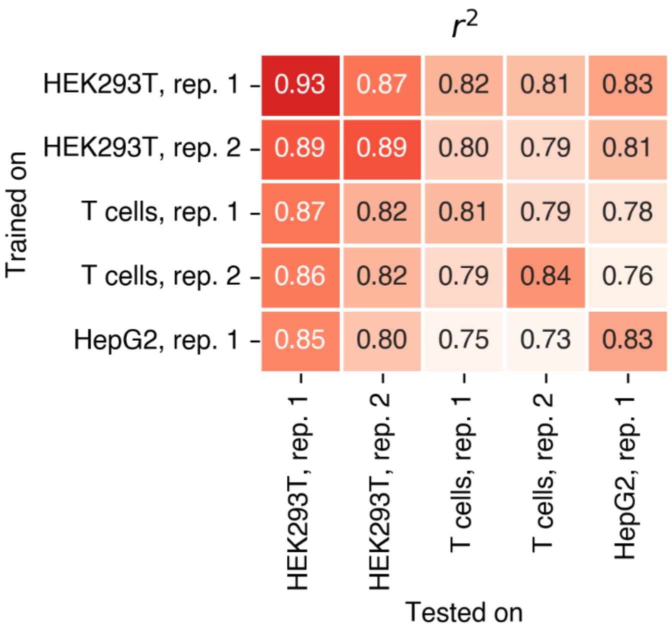

Supplementary Figure 2. Optimus 5-Prime performance when retrained on cell type-specific polysome profiling data. The top 20,000 5'UTR sequences with the highest minimum read coverage across all cell type replicates were separated for testing, and the remaining sequences were used for training. For every cell line and replicate indicated in each row, Optimus 5-Prime was retrained from scratch on the training dataset after filtering for sequences with more than 200 reads. Then, MRL predictions on the test dataset were generated and compared with measurements from each cell line and replicate in each column. Source data are provided as a Source Data file.

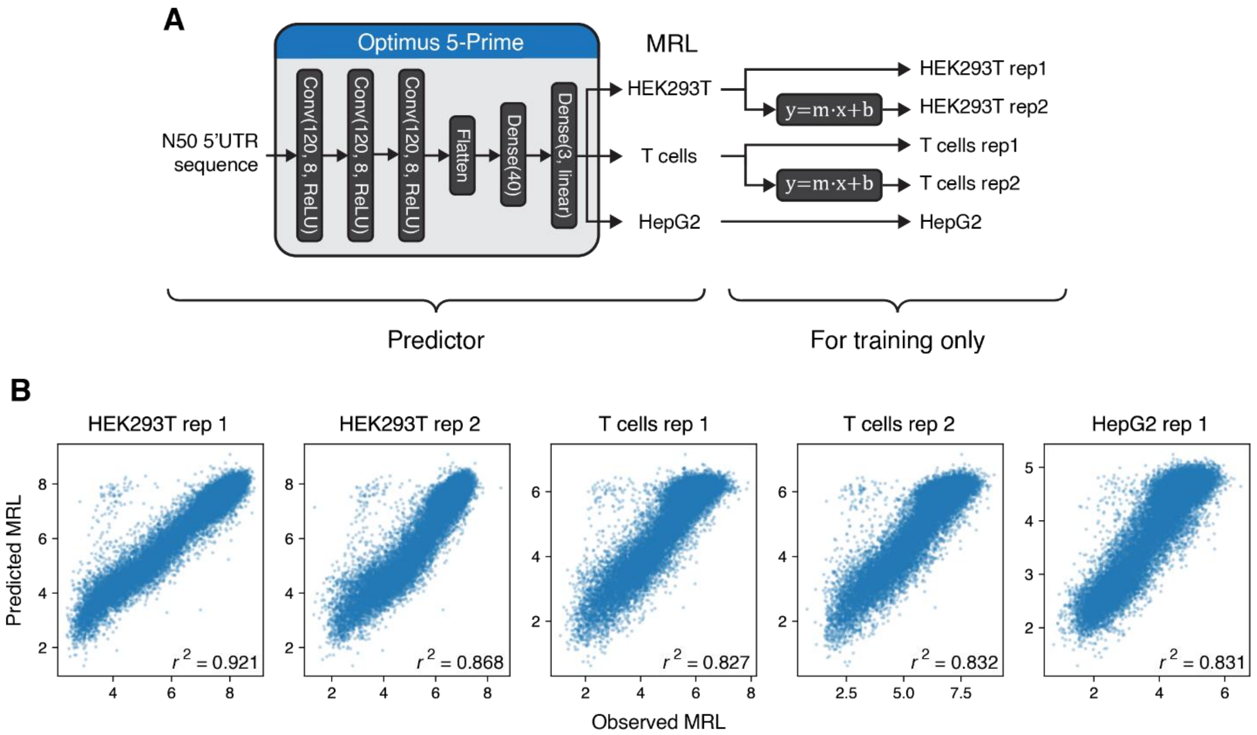

Supplementary Figure 3. Performance of a multi-output version of Optimus 5-Prime. (A) Model diagram. The architecture is the same as the original Optimus 5-Prime, but with a final dense layer with three outputs corresponding to each cell type. During training, we added an additional final layer containing learnable linear scalings to account for systematic bias of replicates of the same cell type. This final layer is not saved with the model after training. **(B)** Model performance when compared to MRL measurements in all cell type replicates, on a held-out test dataset containing 20,000 sequences with the highest minimum read coverage across all replicates. Source data are provided as a Source Data file.

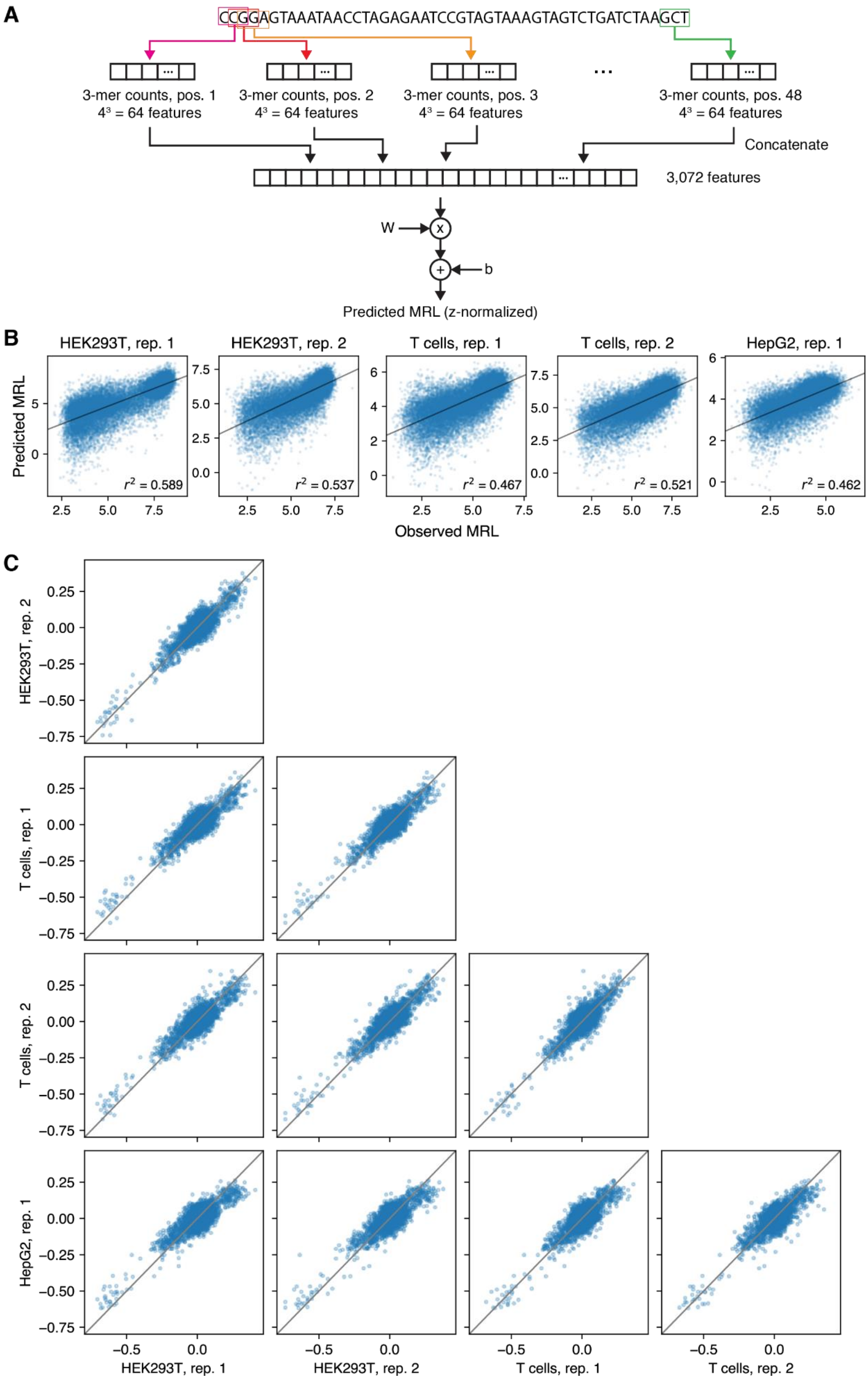
A
CCGGAGTAAATAACCTAGAGAATCCGTAGTAAAGTAGTCTGATCTAAGCT
3-mer counts, pos. 1
4^3 = 64 features
3-mer counts, pos. 2
4^3 = 64 features
3-mer counts, pos. 3
4^3 = 64 features
3-mer counts, pos. 48
4^3 = 64 features
Concatenate
3,072 features
W
b
Predicted MRL (z-normalized)
B
HEK293T, rep. 1
HEK293T, rep. 2
T cells, rep. 1
T cells, rep. 2
HepG2, rep. 1
Predicted MRL
Observed MRL
r^2 = 0.589
r^2 = 0.537
r^2 = 0.467
r^2 = 0.521
r^2 = 0.462
C
HEK293T, rep. 2
T cells, rep. 1
T cells, rep. 2
HepG2, rep. 1
HEK293T, rep. 1
HEK293T, rep. 2
T cells, rep. 1
T cells, rep. 2

Supplementary Figure 4. Analysis of 3-mer with position models across all cell type replicates. (A) Model schematic. Five models, one per cell line and replicate, were trained on z-normalized data using Ridge regression and a regularization coefficient of 1e-5. Regression weights and bias are represented by the 3,072-long vector W and the scalar b. **(B)** Model performance. Models were evaluated on the 20,000 sequences with highest read coverage on each cell line and replicate, which were held out from training. **(C)** Comparison of model parameters (3,072 weights + bias) across all five models. Source data are provided as a Source Data file.

A

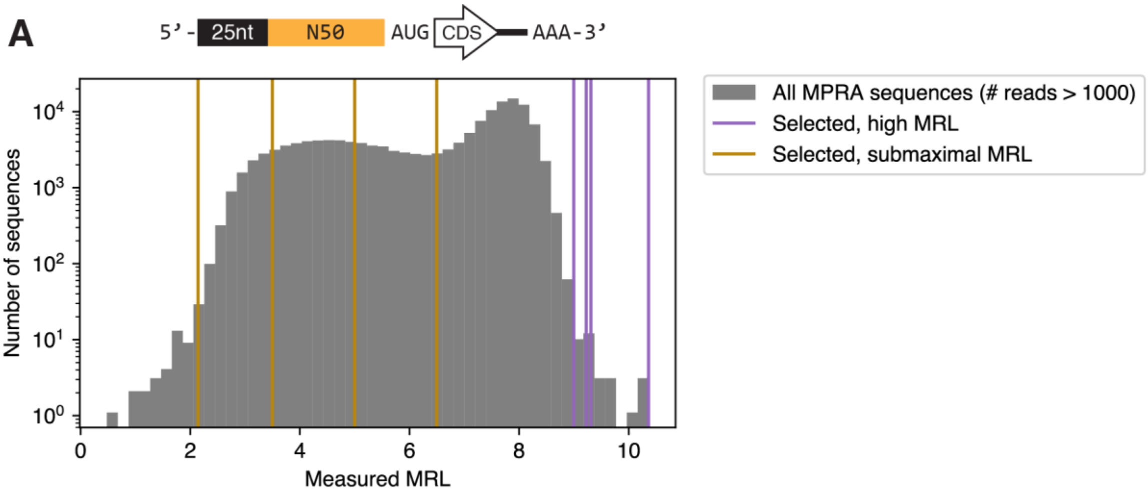

B

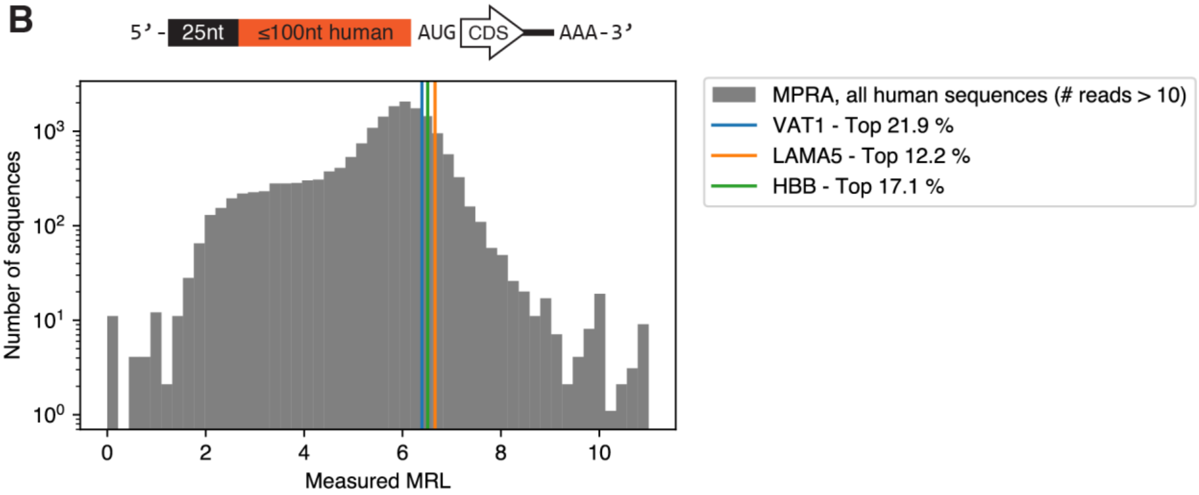

Supplementary Figure 5. Controls for the megaTAL gene editing assays. (A) Selection of controls from the fixed-end MPRA library. For the high MRL control set, starting from the HEK293T MPRA library we excluded sequences with a read count lower than 1,000 or if they contained uATGs or started with TG. The remaining sequences were sorted by MRL, and four from the top twenty were selected. For the submaximal MRL control set, we similarly filtered by read count and excluded sequences with TG at the start, and selected four sequences with MRLs close to 2, 3.5, 5, and 6.5. Top: architecture of the MPRA library. Bottom: Histogram of a high-coverage (# reads > 1,000) subset of the MPRA library, along with the MRLs of all eight selected sequences. **(B)** Selection of controls from a library of short (<=100bp) human 5’UTRs measured in our previous polysome profiling study[1]. Top: architecture of the MPRA library. Bottom: histogram of a subset (16,779 sequences with # reads > 10) of the human 5’UTR library, along with the MRLs of the two selected sequences (***VAT1***, ***LAMA5***) and the hemoglobin beta (HBB) 5’UTR commonly used in mRNA therapeutics. The legend indicates the MRL percentile of these three 5’UTRs compared to the rest of the library. In the megaTAL experiments, human 5’UTR controls did not include the initial constant 25nt, but had a consensus Kozak sequence (GCCACC) appended at their 3’ end. See **Supplementary Data 1** for full sequences. **Source data are provided as a Source Data file.**

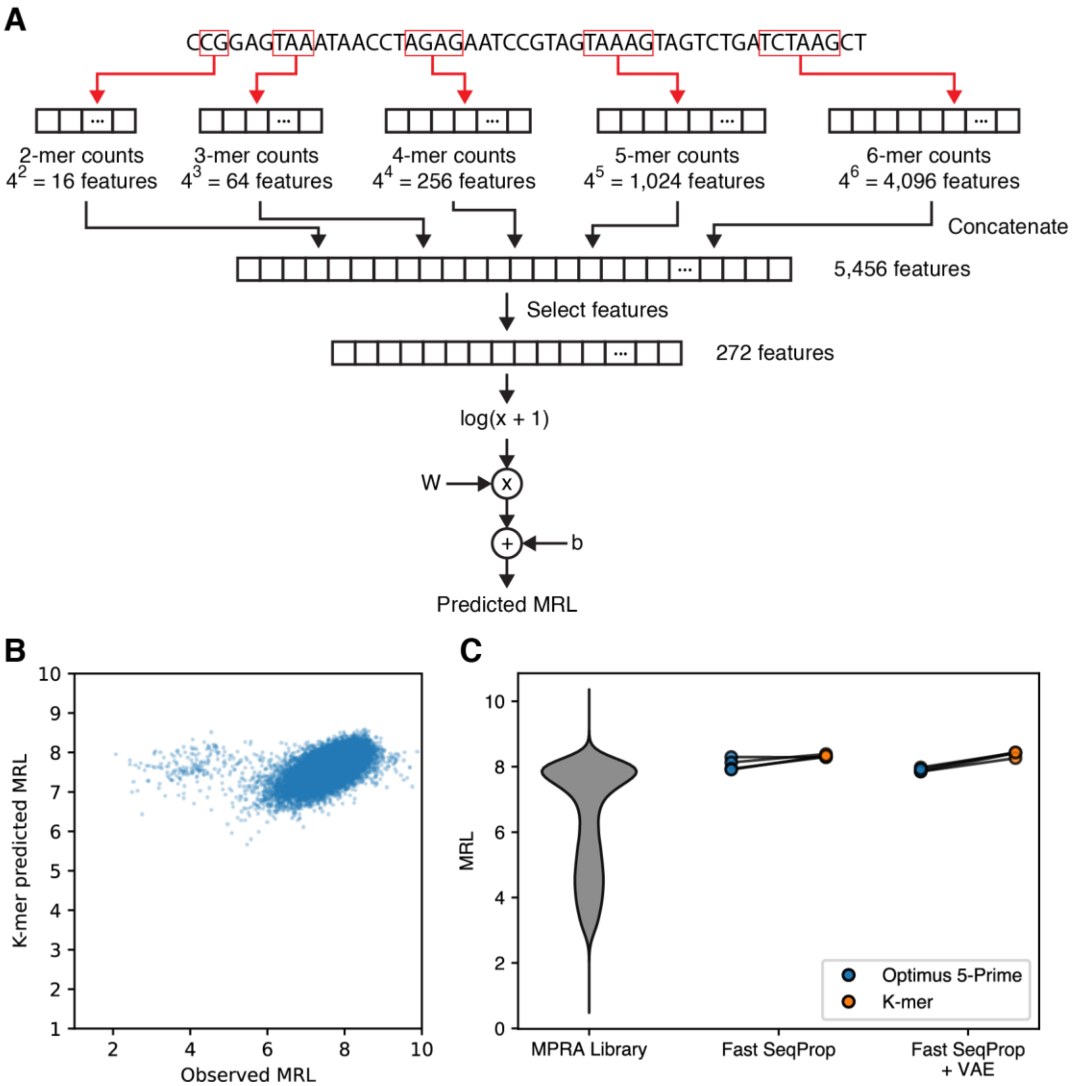

Supplementary Figure 6. Using a linear k-mer model to validate fixed-end 50nt-long Fast SeqProp designs. (A) k-mer model architecture. W is a 272-long weight vector, and b is a scalar bias. See **Methods** for a description of the model and training procedure. **(B)** Observed vs. predicted MRL on a held-out set of 25,931 5'UTRs with no uAUG and greater than 250 reads. Pearson r = 0.5213 **(C)** Comparison of predicted MRL for the four sequences designed via Fast SeqProp and the four sequences designed via Fast SeqProp with VAE regularization, when using Optimus 5-Prime or the k-mer model from panel **(A)**. A violin plot of the entire MPRA library is shown on the left for comparison. K-mer model predictions from the designed sequences are within the top 1% compared to equivalent predictions on the entire test set shown in **(B)**. Source data are provided as a Source Data file.

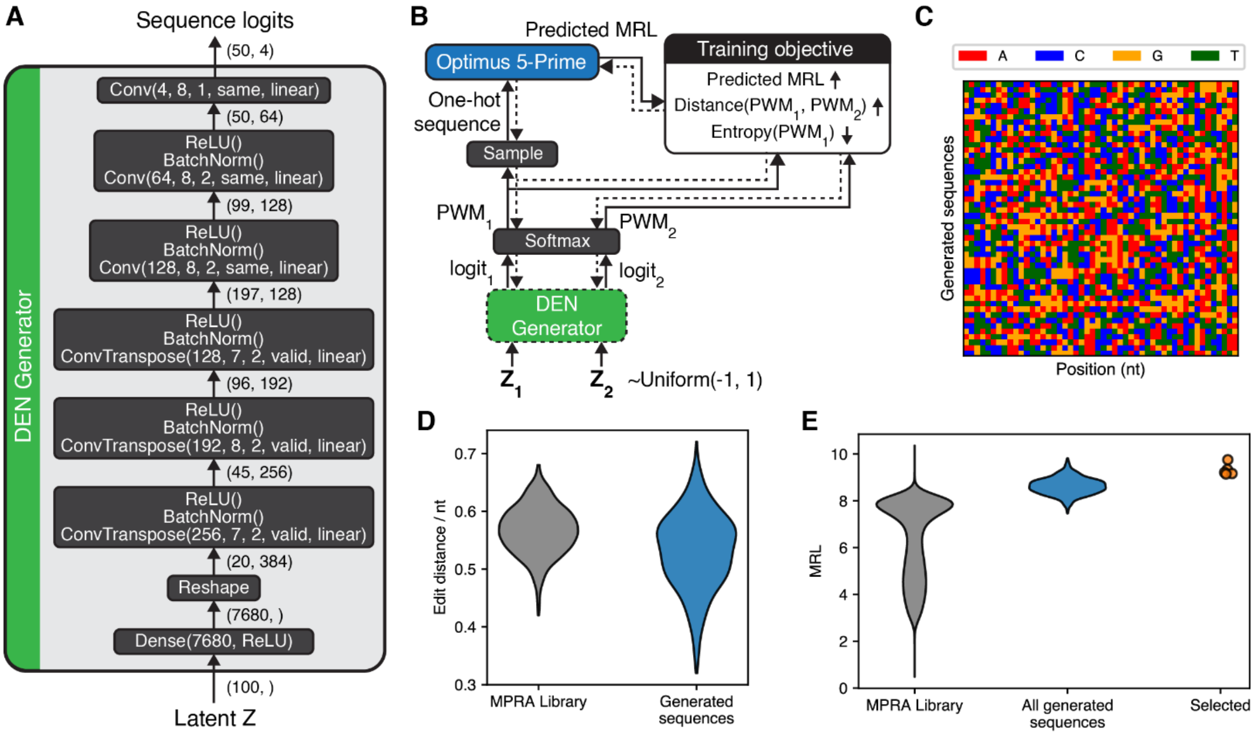

Supplementary Figure 7. 50nt 5'UTR design using Optimus 5-Prime and a Deep Exploration Network (DEN). (A) Architecture of the DEN generator network, which takes a continuous-valued 100-dimensional latent vector and returns a 50x4-dimesional continuous-valued logit representing a sequence. Convolutional and Transpose convolutional layers are represented as $\mathrm{Conv}(F, W, S, P, A)$ and $\mathrm{ConvTranspose}(F, W, S, P, A)$, where F and W are the number and size of convolutional filters, S is the stride, P is the padding, and A is the activation function. **(B)** DEN training schematic. Only the DEN generator's weights are optimized via gradient descent. As the predictor, we used a retrained version of Optimus 5-Prime, initially trained on the fixed 50nt MPRA data and finetuned on sequences we previously designed to maximize MRL that ultimately underperformed[1]. At any iteration, two sequence logits are generated from two uniformly random seed vectors. Optimus 5-Prime is used to obtain an MRL prediction from a one hot-encoded sequence sampled from logit_1. The training objective to minimize is the weighted sum of the following components: 1) a fitness component set to $-\mathrm{MRL}$, 2) a similarity component calculated from both PWMs as follows $\max\left(0, \mathrm{CosineSimilarity}(\mathrm{PWM}_1, \mathrm{PWM}_2) - \mathrm{margin}_{\mathrm{similarity}}\right)$ and 3) an entropy component set as the Shannon Entropy of PWM_1 up to a margin $\max\left(0, \mathrm{margin}_{\mathrm{entropy}} - (2 - \mathrm{entropy})\right)$ to ensure PWM_1 is close to a one hot-encoded sequence. Weights for the fitness, similarity, and entropy components of the loss function were 0.1, 5, and 1. Margin values for the similarity and entropy terms were 0.3 and 1.8. Training was performed for 250 epochs. **(C)** Colormap representation of 50 5'UTR sequences randomly chosen from the 1,024 generated by the trained DEN. Each row represents a separate sequence, and color indicates nucleotide identity. **(D)** Distribution of edit distances per nucleotide, for 500 random pairs chosen from the MPRA library (left) or the 1,024 sequences generated by the DEN. **(E)** Distribution of MRLs measured from the MPRA library (left), or predicted by Optimus 5-Prime on all 1,024 DEN-generated sequences (middle) and the four selected sequences from this set (right). Source data are provided as a Source Data file.

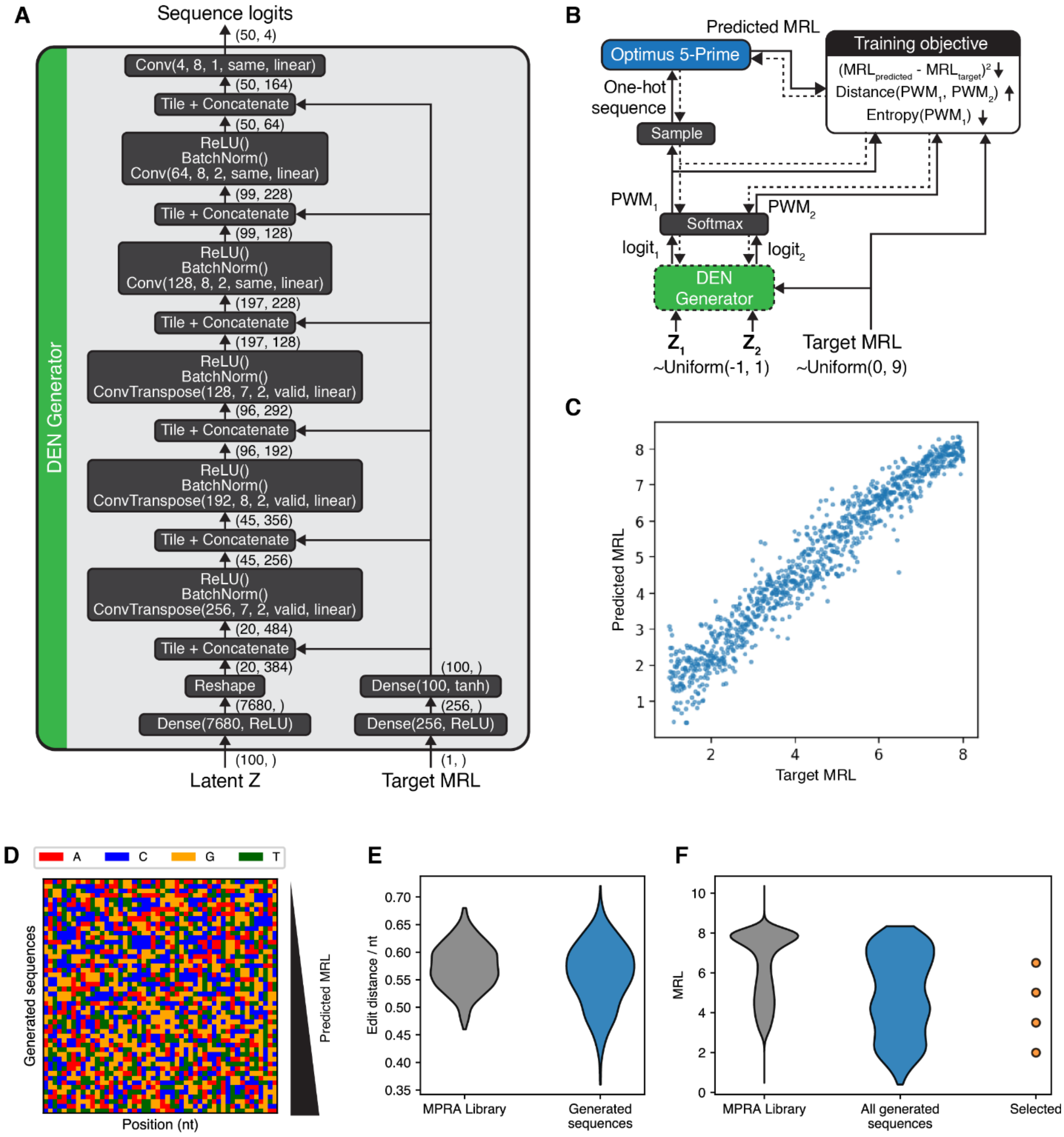

Supplementary Figure 8. Design of 5'UTR sequences of varying MRLs using Optimus 5-Prime and an inverse regression-type Deep Exploration Network (DEN). (A) Architecture of the DEN generator network. Compared to **Supplementary Figure 7**, this network has an additional input representing the target MRL of the 5'UTR to be generated. Additionally, after being processed by two dense layers, this input is concatenated to the input of each convolutional layer. **(B)** DEN training schematic. Compared to **Supplementary Figure 7**, we additionally sample target MRL values from a uniformly random distribution during training, and set the fitness loss to the squared difference between the target and predicted MRL. Weights for the fitness, similarity, and entropy components of the loss function were 0.2, 5, and 1. Margin values for the similarity and entropy terms were 0.3 and 1.8. Training was performed for 100 epochs. **(C)** After DEN training, 1,024 5'UTR sequences covering a range of target MRLs were generated and compared to the MRL predicted by Optimus 5-Prime. **(D)** Colormap representation of 50 5'UTR sequences randomly chosen from the 1,024 generated by the trained DEN, sorted by predicted MRL. Each row represents a separate sequence, and color indicates nucleotide identity. **(E)** Distribution of edit distances per nucleotide, for 500 random pairs chosen from the MPRA library (left) or the 1,024 sequences generated by the DEN. **(F)** Distribution of MRLs measured from the MPRA

library (left), or predicted by Optimus 5-Prime on all 1,024 DEN-generated sequences (middle) and the four selected sequences from this set (right). Source data are provided as a Source Data file.

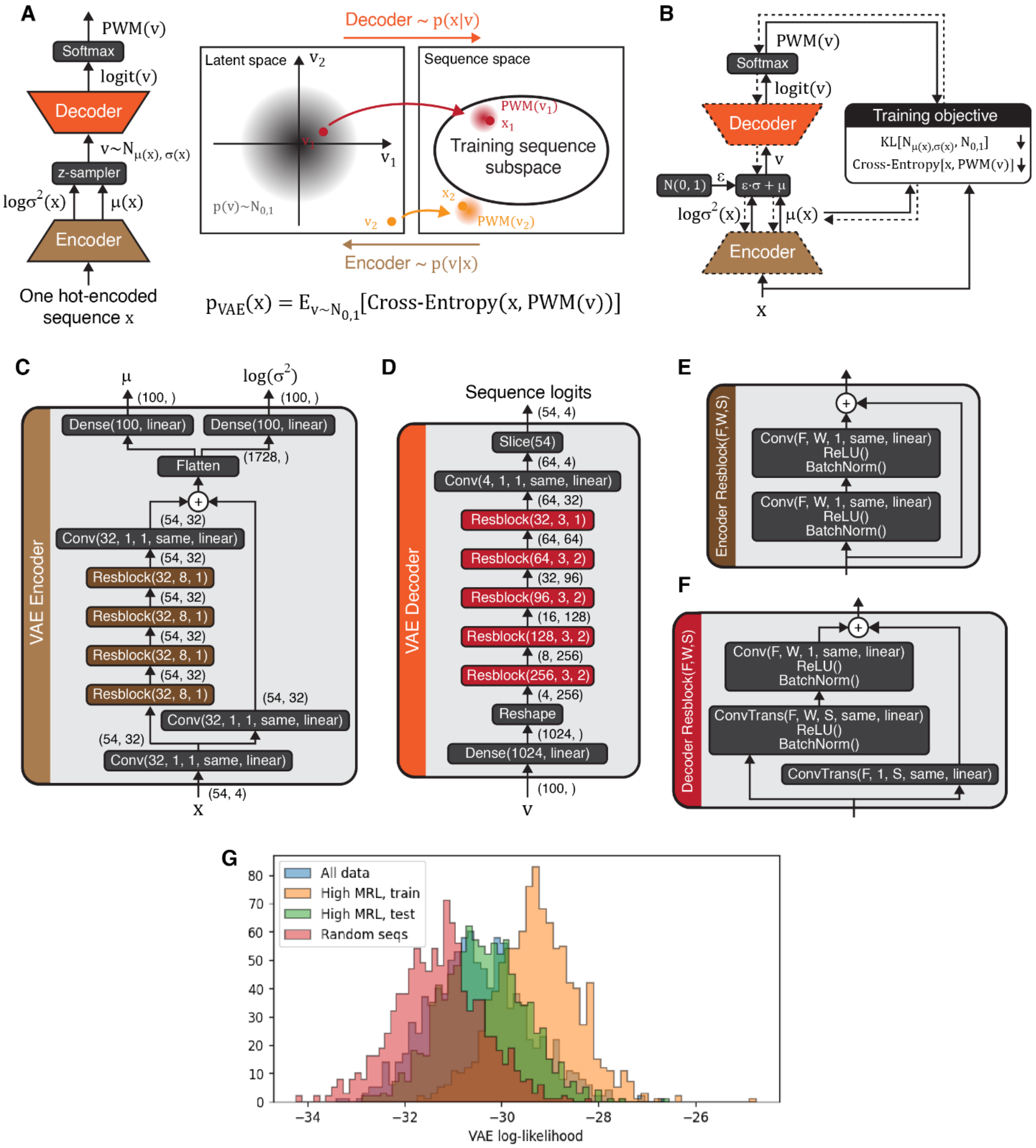

Supplementary Figure 9. Variational Autoencoder (VAE) to estimate the likelihood of 5'UTR sequences in the fixed-end 50nt MPRA. (A) Schematic of a VAE structure and function. In the VAE framework, a sequence x originates from sampling a latent vector v from a continuous prior distribution $p(v) \sim N(0,1)$, followed by sampling x from the likelihood $p(x|v)$. Sequences in the dataset used for VAE training correspond to latent vectors close to 0 and therefore more likely under the prior. Two neural networks allow (probabilistic) conversion between the sequence and the latent space. On one hand, an encoder accepts a one hot-encoded x and returns the mean μ and log variance $\log(\sigma^2)$ of a normal distribution corresponding to $p(v|x)$. Conversely, a decoder converts a latent vector into a sequence logit, which can be converted into a PWM encoding $p(x|v)$. Conceptually, the marginal probability of a sequence $p(x) \cong p_{VAE}(x)$ is the expected cross-entropy ("distance") between the x and the output $\mathrm{PWM}(v)$, when v is sampled from $p(v) = N(0,1)$. In practice, it is more efficient to sample v from $N(\mu(x), \sigma^2(x))$ and use a correction factor to account for the different distribution (importance sampling). For implementation details, see[2]. **(B)** During

VAE training, encoder and decoder weights are updated via gradient descent to minimize the KL-divergence between $N(0,1)$ and $N(\mu(x), \sigma^2(x))$, as well as the cross-entropy between x and $\mathrm{PWM}(v)$, for all x in the training set. For a more comprehensive description of VAE training, see[2]. **(C-F)** Architecture of the Encoder network **(C)**, decoder network **(D)**, and the residual blocks used in the encoder **(E)** and decoder **(F)**. Convolutional and transpose convolutional layers are represented as $\mathrm{Conv}(F, W, S, P, A)$ and $\mathrm{ConvTrans}(F, W, S, P, A)$, where F and W are the number and size of convolutional filters, S is the stride, P is the padding, and A is the activation function. For the 50nt VAE, a 54nt-long sequence is used where the last 4 bases are masked out with zeros. **(G)** Likelihood of different sequence sets under a VAE trained on high MRL 5'UTR sequences. Histograms were generated from 1,000 sequences randomly selected from the full MPRA library, the VAE training and testing sets, or randomly generated *in silico*. Source data are provided as a Source Data file.

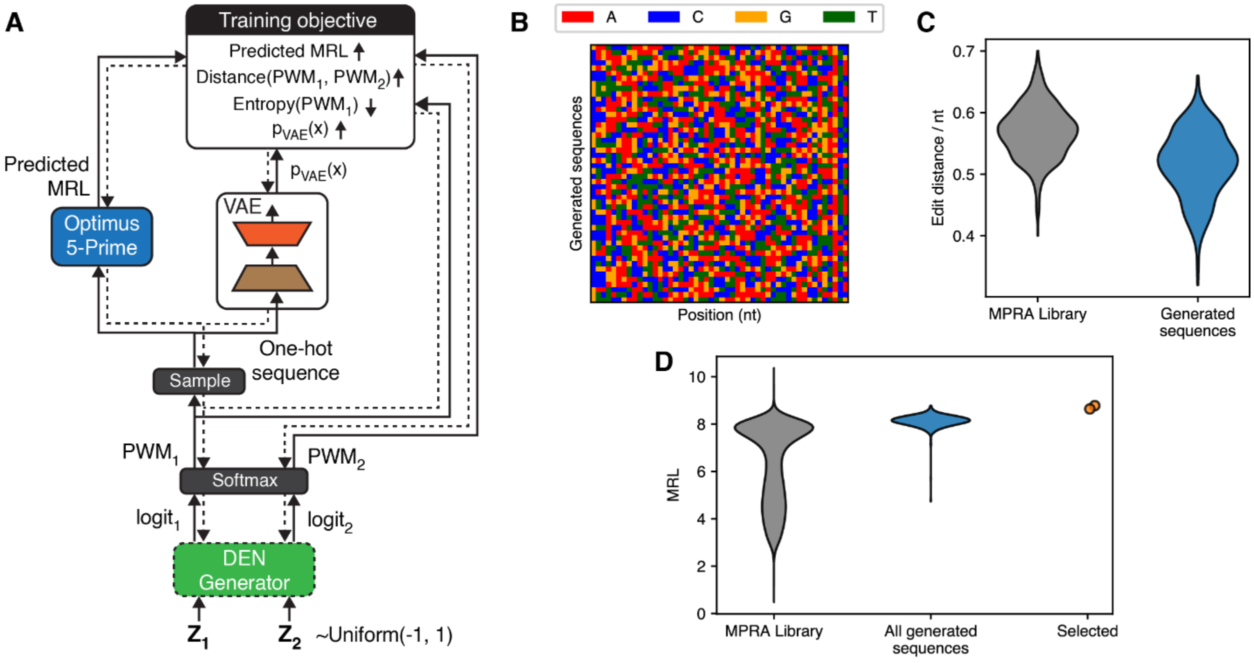

Supplementary Figure 10. 50nt 5'UTR design using Optimus 5-Prime, a Deep Exploration Network (DEN), and VAE regularization. (A) DEN training schematic. Compared to **Supplementary Figure 7**, we here used a VAE pretrained as shown in **Supplementary Figure 9** to estimate the marginal $p_{VAE}(x)$ of a generated sequence x, and we add a VAE component to the loss function set to $\max(0, \text{margin}_{VAE} - \log(p_{VAE}(x)))$. Weights for the fitness, similarity, entropy, and VAE components of the loss function were 0.1, 5, 1, and 0.5. Margin values for the similarity, entropy, and VAE terms were 0.3, 1.8, and -30. Training was performed for 100 epochs. **(B)** Colormap representation of 50 5'UTR sequences randomly chosen from the 1,024 generated by the trained DEN. Each row represents a separate sequence, and color indicates nucleotide identity. **(C)** Distribution of edit distances per nucleotide, for 500 random pairs chosen from the MPRA library (left) or the 1,024 sequences generated by the DEN. **(D)** Distribution of MRLs measured from the MPRA library (left), or predicted by Optimus 5-Prime on all 1,024 DEN-generated sequences (middle) and the two selected sequences from this set (right). Source data are provided as a Source Data file.

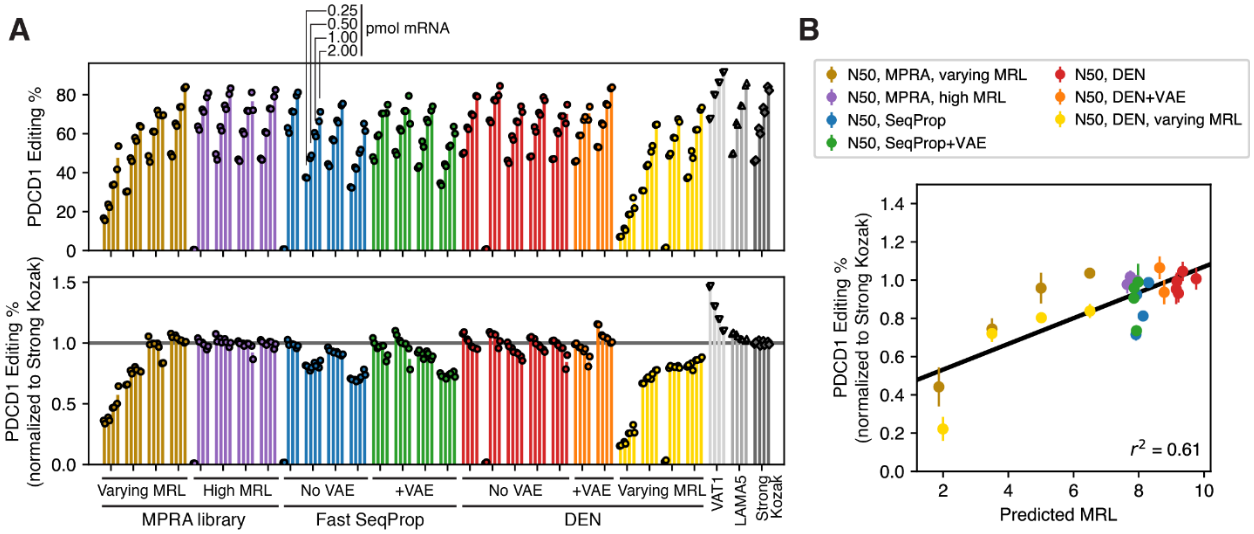

Supplementary Figure 11. Performance of 50nt 5'UTR designs on the *PDCD1* megaTAL. (A) Editing efficiencies for mRNAs with a megaTAL targeting the *PDCD1* gene, for 30 different 5'UTR including designs and controls. Top: absolute editing efficiencies. Bottom: editing efficiencies normalized to the Strong Kozak control. Analogous to **Figure 2C** but with the *PDCD1* megaTAL instead of *TGFBR2*. Editing efficiencies for the first High MRL MPRA control, the first No VAE Fast SeqProp design, the second No VAE DEN design, and the third Varying MRL DEN design were close to zero only at a dosage of 0.25 pmol mRNA, and were deemed to be the result of experimental error and excluded from subsequent analysis. **(B)** Kozak-normalized editing efficiency of the *PDCD1* megaTAL vs. Optimus 5-Prime predicted MRL for all designed and MPRA control 5'UTRs. Each marker and error bar represent the mean and standard deviation of the Kozak-normalized editing efficiencies, as shown in the bottom panel of **(A)**, across all mRNA dosages for a particular 5'UTR (n=8 for most 5'UTRs: 2 biological replicates and 4 dosages per replicate. The 0.25 pmol dosage was excluded for four sequences as described above, therefore n=6 for these 5'UTRs only). Analogous to **Figure 2E** but with the *PDCD1* megaTAL instead of *TGFBR2*. Source data are provided as a Source Data file.

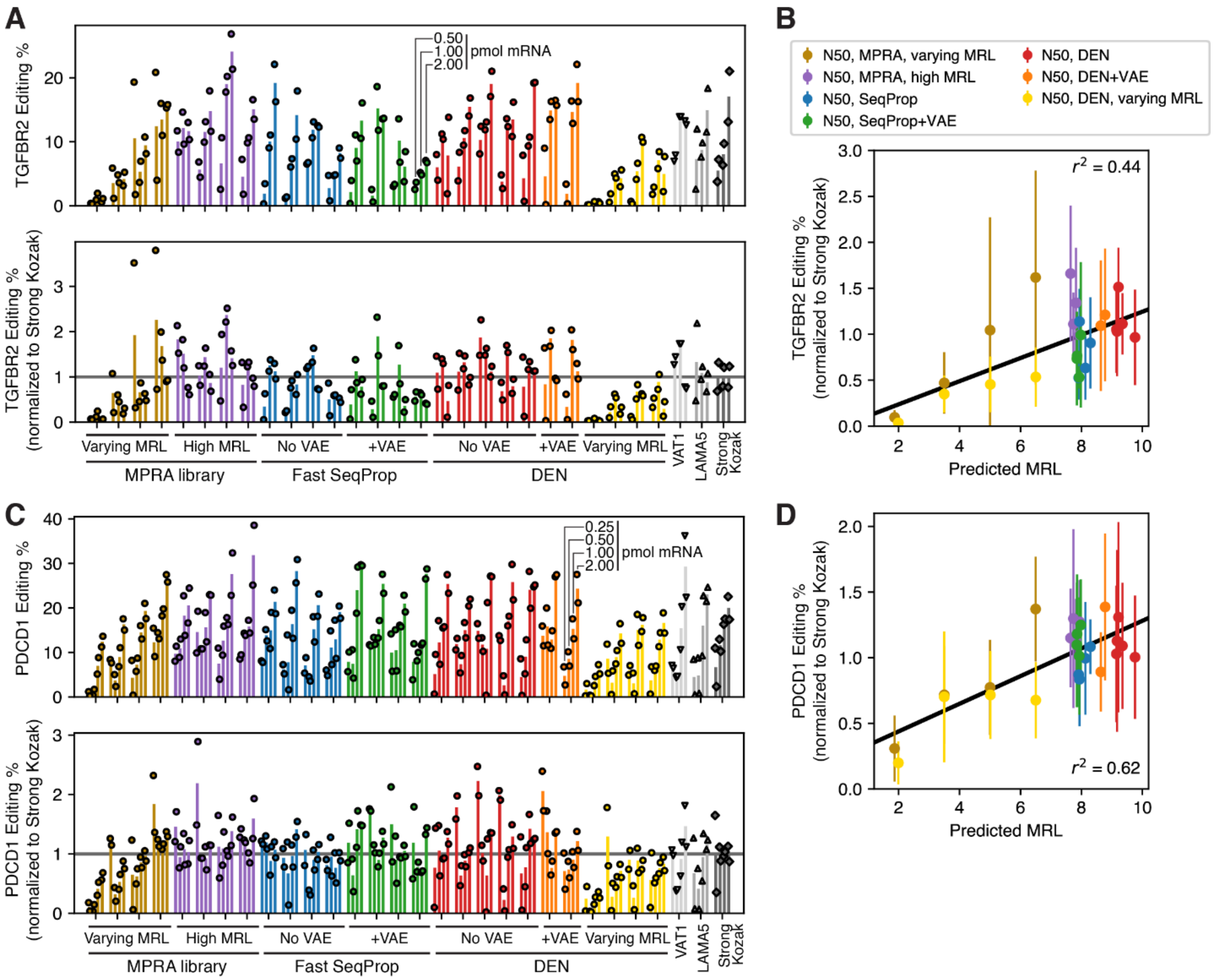

Supplementary Figure 12. Performance of 50nt 5'UTR designs on the *TGFBR2* and *PDCD1* megaTALs in HepG2. (A and C) Editing efficiencies for mRNAs with a megaTAL targeting the *TGFBR2* **(A)** or *PDCD1* **(C)** genes in HepG2 cells, for 30 different 5'UTR including designs and controls. Top: absolute editing efficiencies. Bottom: editing efficiencies normalized to the Strong Kozak control. Analogous to **Figure 2C** and **Supplementary Figure 11A** but using HepG2 cells instead of K562. Only three mRNA dosage levels were evaluated for *TGFBR2*. **(B and D)** Kozak-normalized editing efficiencies of the *TGFBR2* **(B)** and *PDCD1* **(D)** megaTALs vs. Optimus 5-Prime predicted MRL for all designed and MPRA control 5'UTRs. In **(B)** and **(D)**, each marker and error bar represent the mean and standard deviation of the Kozak-normalized editing efficiencies, as shown in the bottom panels of **(A)** and **(C)**, across all mRNA dosages for a particular 5'UTR (n=8: 2 biological replicates at 4 dosages per replicate). Analogous to **Figure 2E** and **Supplementary Figure 11B** but using HepG2 cells instead of K562. Source data are provided as a Source Data file.

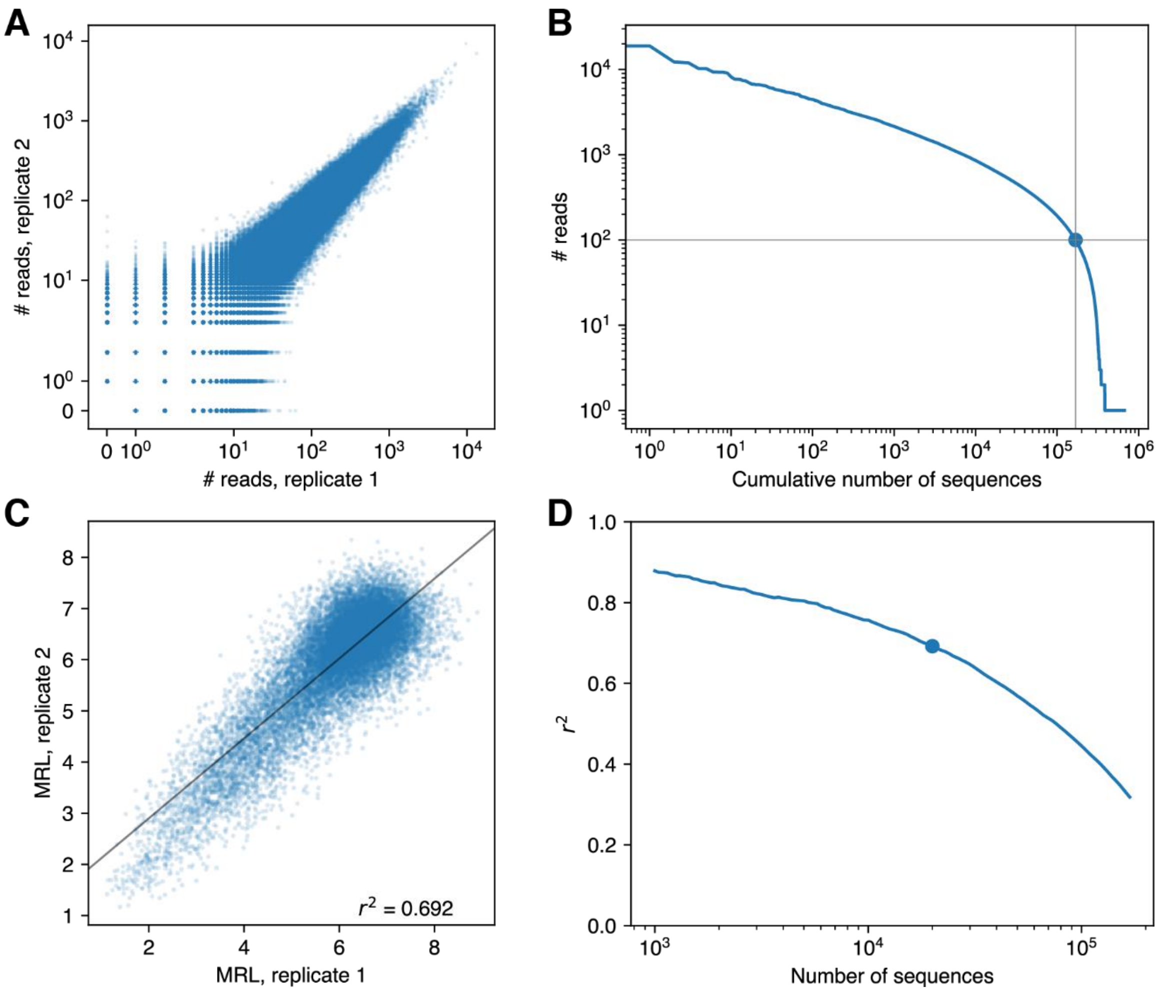

Supplementary Figure 13. Basic analysis of random-end N25 MPRA library. (A) Sequencing read coverage for all sequences across two biological replicates. **(B)** Number of sequences resulting from a given cutoff on the total number of reads per sequence across both replicates. Marker indicates 168,297 sequences with at least 100 reads. **(C)** MRL correlation across replicates, for the top 20,000 sequences by read coverage. Black line represents a regression line. **(D)** r^2 as a function of the number of sequences used. Sequences were sorted by the total number of reads across both replicates in descending order. Then, the top x sequences (x axis) were used to calculate a corresponding r^2 value (y axis). The large marker indicates the number of sequences and r^2 in **(C)**. Source data are provided as a Source Data file.

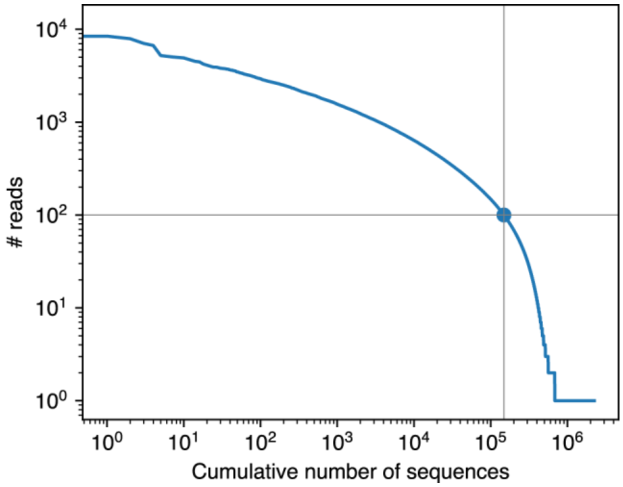

Supplementary Figure 14. Read coverage as a function of the number of sequences retained in the random-end N50 MPRA. Marker indicates 57,165 sequences with at least 100 reads. Source data are provided as a Source Data file.

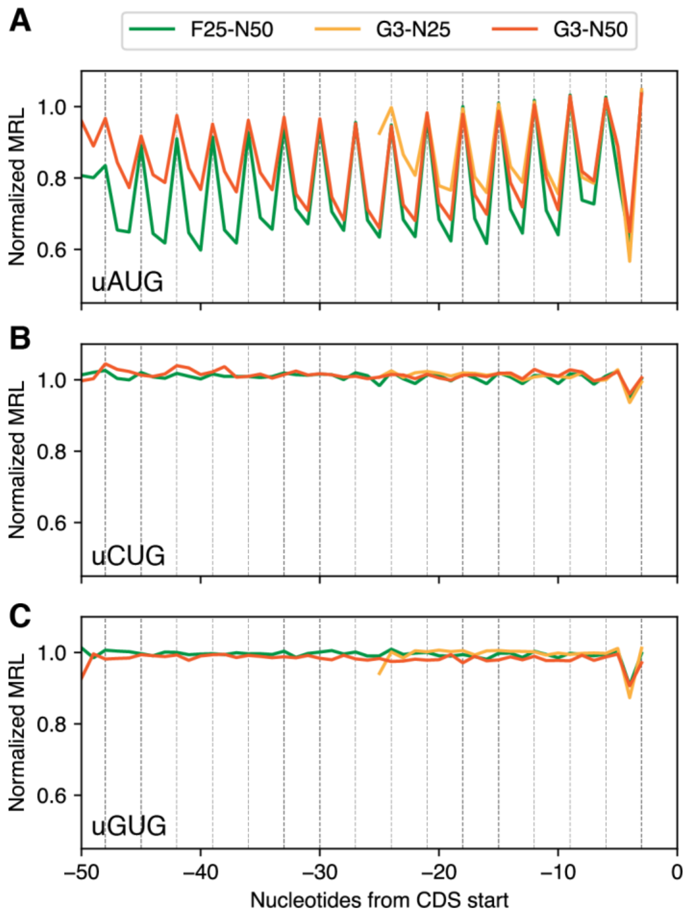

Supplementary Figure 15. Effects of upstream start codons on MRL in three MPRA libraries. Median MRL of all sequences containing a uAUG **(A)**, uCUG **(B)**, and uGUG **(C)** at the indicated position from the start of the EGFP ORF, for the 25nt- (yellow) or 50nt-long (orange) randomized 5'UTR libraries, as well as our previous fixed-end 50nt library (green). MRL was normalized to the median of each library. **(A)** is identical to **Figure 3B** but aligned to the EGFP start codon instead of the start of the transcript. Source data are provided as a Source Data file.

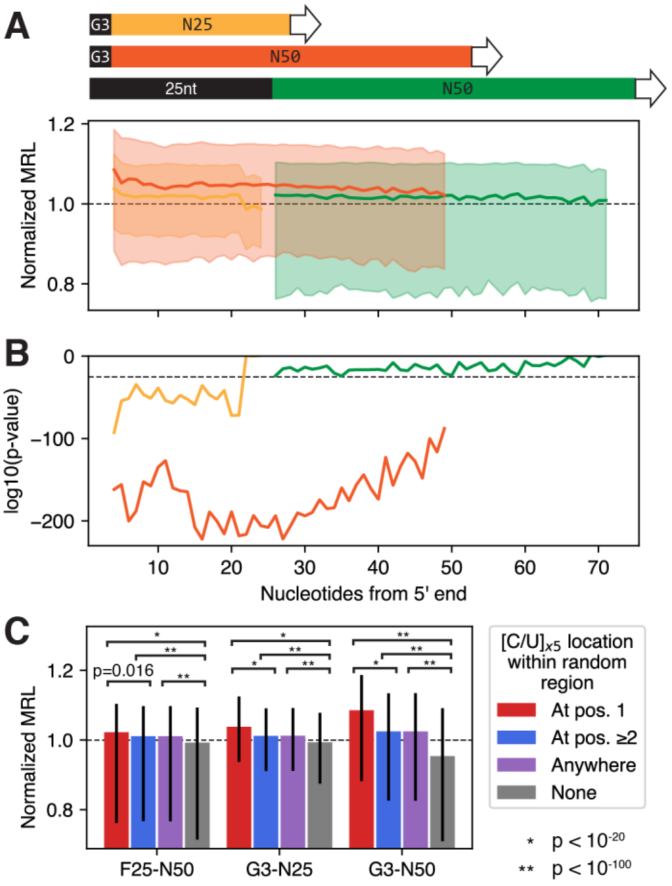

Supplementary Figure 16. Detailed analysis of the effects of 5'UTR polypyrimidine tracts on MRL in three MPRA libraries. (A) Median MRL (solid lines) and interquartile range (shaded regions) of all sequences containing 5nt-long polypyrimidine (C or U) tract at the indicated position from the start of the transcript. Identical to **Figure 3C** but with the interquartile range indicated. **(B)** Bonferroni-corrected p-values from a Mann Whitney U test of medians between the MRL of sequences that contain a 5nt-long polypyrimidine tract at the indicated position versus sequences that do not contain 5nt-long polypyrimidine tracts at all. Horizontal bar indicates $p = 10^{-25}$. **(C)** MRL of library sequences containing a 5nt-long polypyrimidine tract (C or U) within the random region, starting at position 1 (red bars), 2 or after (blue bars), anywhere (purple bars), or none at all (gray bars). MRLs were normalized to the median of each library as in **(A)**. Bars indicate the normalized median MRL within each group. Error bars indicate the interquartile range. Horizontal brackets indicate Bonferroni-corrected p-values from a Mann Whitney U test of medians between the MRLs in each group. *: $p < 10^{-20}$, **: $p < 10^{-100}$. Source data are provided as a Source Data file.

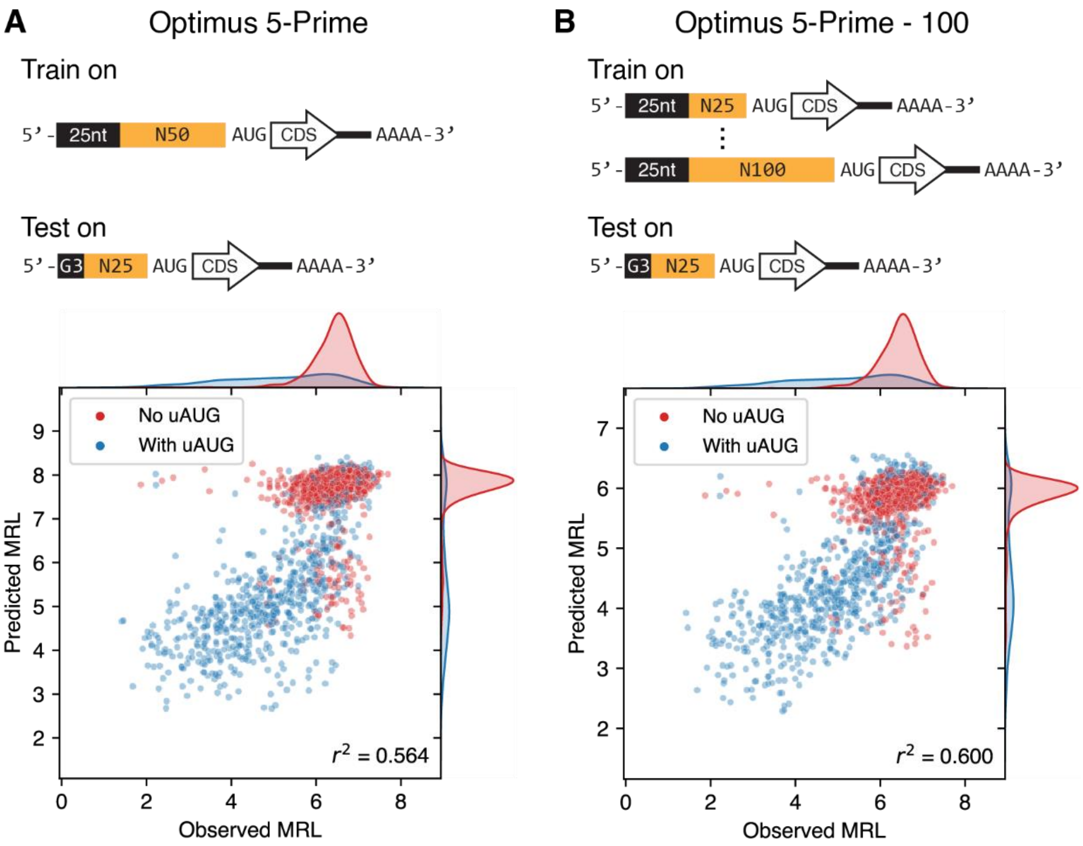

Supplementary Figure 17. Performance of previously developed Optimus 5-Prime models on the random-end 25nt library. (A) Performance of the original Optimus 5-Prime trained on the fixed-end 50nt 5’UTR library. **(B)** Performance of Optimus 5-Prime - 100[1], a model with the same architecture as the original Optimus 5-Prime but trained on a 5’UTR library with a fixed 25nt segment followed by a variable region between 25 and 100nt long. As in **Figure 3E**, these models were evaluated against a test dataset comprised of the 2,000 sequences with the highest read coverage in the random-end 25nt MPRA library. Source data are provided as a Source Data file.

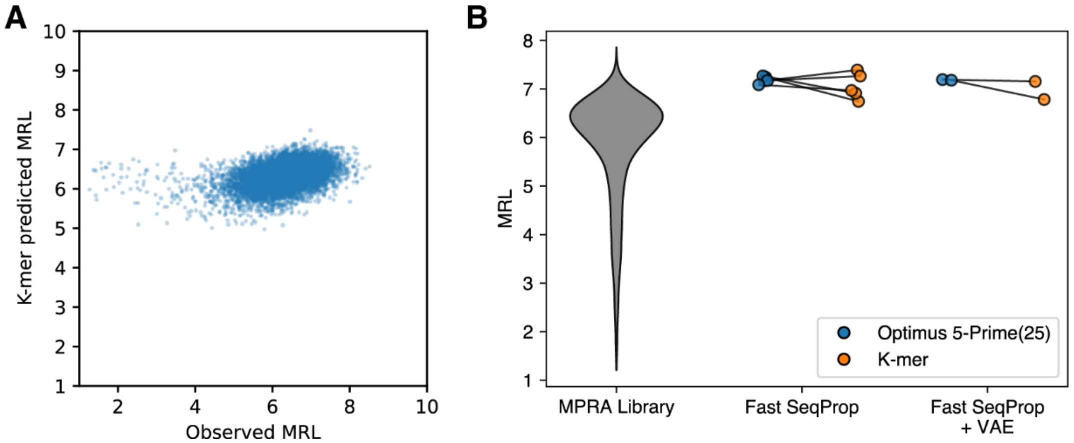

Supplementary Figure 18. Using a linear k-mer model to validate 25nt-long Fast SeqProp designs. See **Methods** for training and model details. **(A)** Observed vs. predicted MRL on a held-out set of 11,349 sequences with no uAUG and greater than 200 reads. Pearson r = 0.4094. **(B)** Comparison of predicted MRL for the five sequences designed via Fast SeqProp and the two sequences designed via Fast SeqProp with VAE regularization, when using Optimus 5-Prime(25) or the k-mer predictors. A violin of the entire MPRA library is shown on the left for comparison. Compared to k-mer model predictions on the entire test set shown in **(A)**, most predictions on the designed sequences are within the top 1%. The exceptions are two sequences designed without VAE regularization, which are within the top 1.2% and 6.3%, and one VAE-regularized design, which is within the top 4.5%. Source data are provided as a Source Data file.

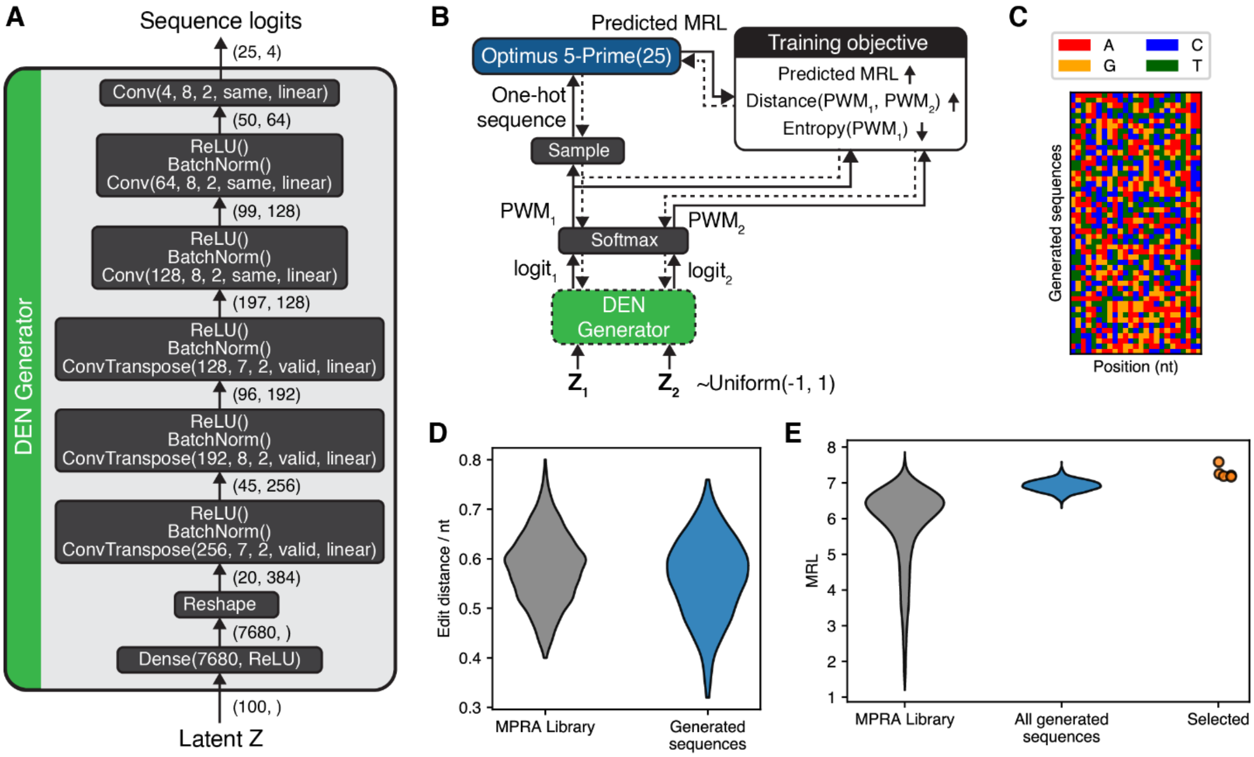

Supplementary Figure 19. 25nt 5'UTR design using Optimus 5-Prime(25) and Deep Exploration Networks. (A) Architecture of the DEN generator network, which takes a continuous-valued 100-dimensional latent vector and returns a 25x4-dimesional continuous-valued logit representing a sequence. Convolutional and Transpose convolutional layers are represented as $\mathrm{Conv}(F, W, S, P, A)$ and $\mathrm{ConvTranspose}(F, W, S, P, A)$, where F and W are the number and size of convolutional filters, S is the stride, P is the padding, and A is the activation function. **(B)** DEN training schematic. Training was performed as described in **Supplementary Figure 7** but with Optimus 5-Prime(25) (**Figure 3**) as the predictor. Weights for the fitness, similarity, and entropy components of the loss function were 0.35, 5, and 1. Margin values for the similarity and entropy terms were 0.3 and 1.8. Training was performed for 100 epochs. **(C)** Colormap representation of 50 5'UTR sequences randomly chosen from the 1,024 generated by the trained DEN. Each row represents a separate sequence, and color indicates nucleotide identity. **(D)** Distribution of edit distances per nucleotide, for 500 random pairs chosen from the 25nt random-end MPRA library (left) or the 1,024 sequences generated by the DEN. **(E)** Distribution of MRLs measured from the MPRA library (left), or predicted by Optimus 5-Prime(25) on all 1,024 DEN-generated sequences (middle) and the four selected sequences from this set (right). Source data are provided as a Source Data file.

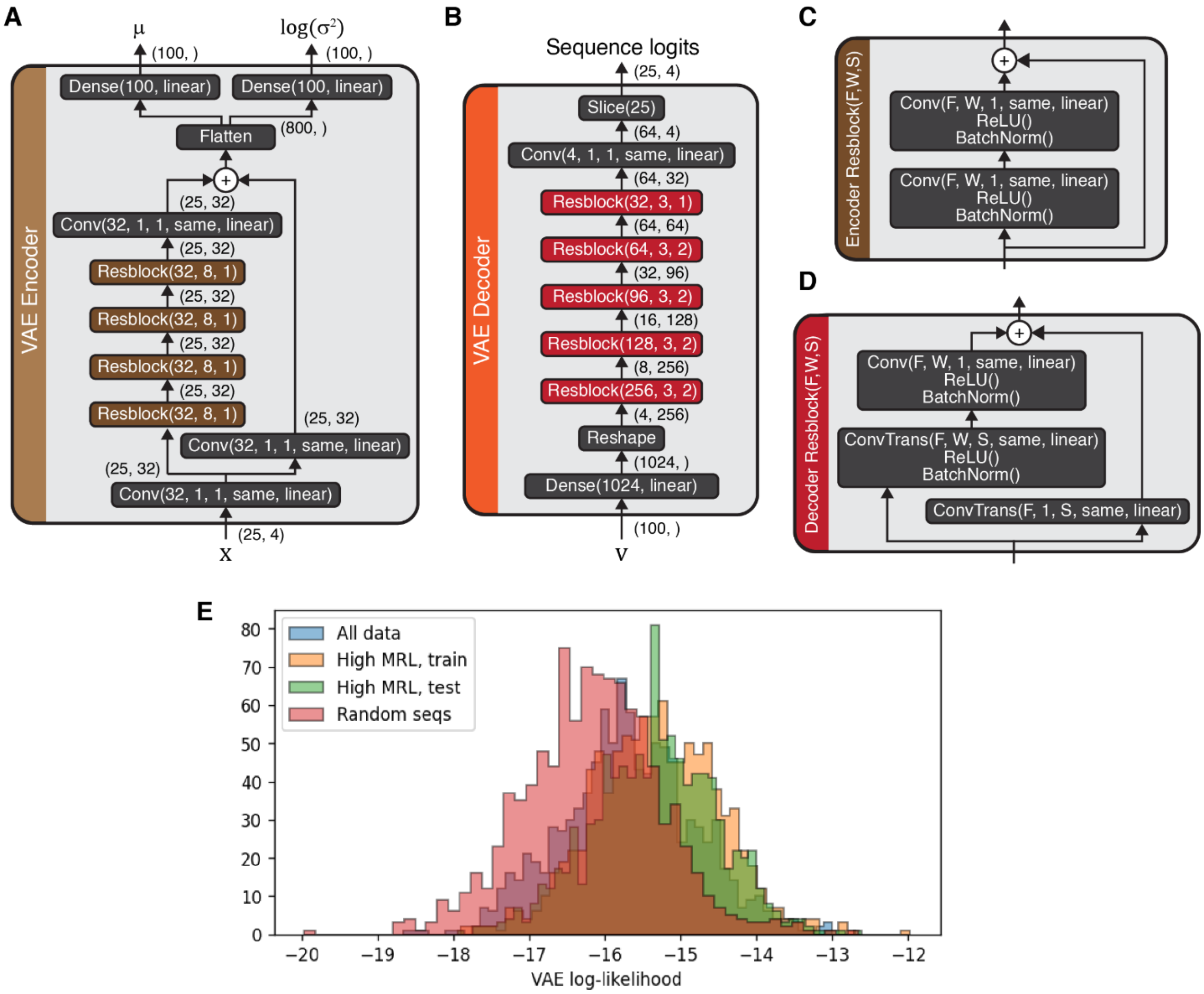

Supplementary Figure 20. Variational Autoencoder (VAE) to estimate the likelihood of 5'UTR sequences in the 25nt random-end MPRA. The general VAE architecture and training scheme is identical to those in **Supplementary Figure 9A** and **B**. **(A-D)** Architecture of the Encoder network **(A)**, decoder network **(B)**, and the residual blocks used in the encoder **(C)** and decoder **(D)**. Convolutional and transpose convolutional layers are represented as $\mathrm{Conv}(F, W, S, P, A)$ and $\mathrm{ConvTrans}(F, W, S, P, A)$, where F and W are the number and size of convolutional filters, S is the stride, P is the padding, and A is the activation function. **(E)** Likelihood of different sequence sets under a VAE trained on high MRL 5'UTR sequences. Histograms were generated from 1,000 sequences randomly selected from the full MPRA library, the VAE training and testing sets, or randomly generated in silico. Source data are provided as a Source Data file.

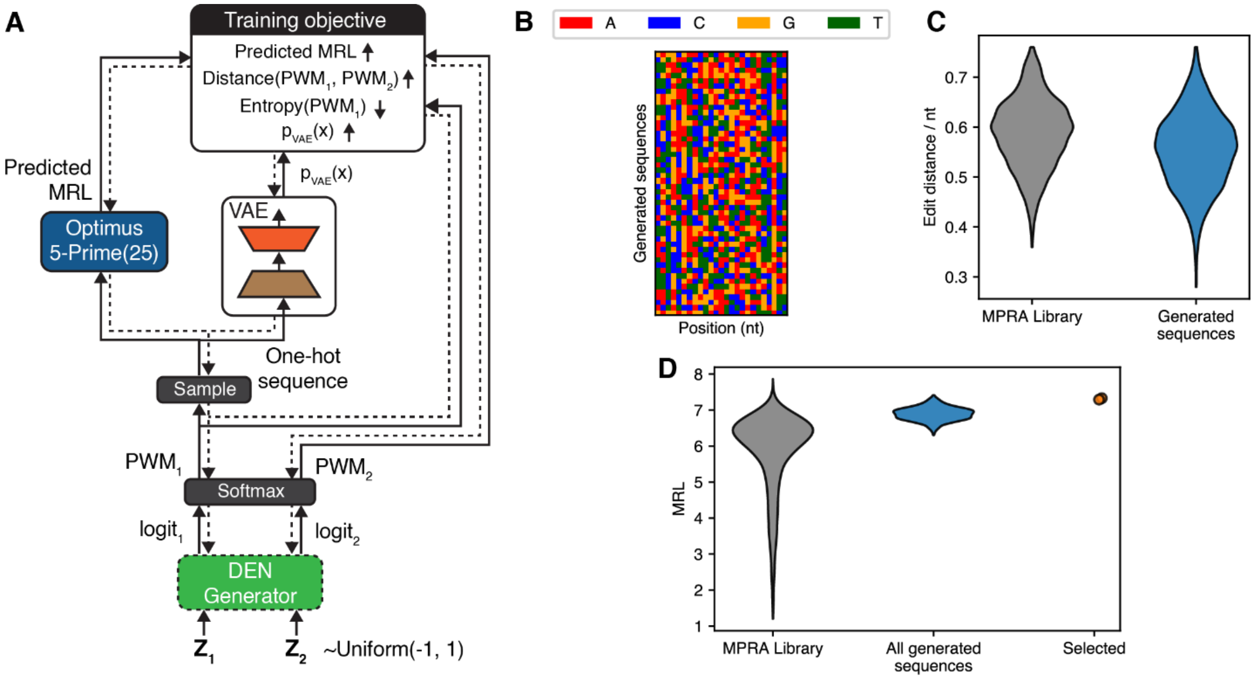

Supplementary Figure 21. 25nt-long 5'UTR design using Optimus 5-Prime(25), a Deep Exploration Network (DEN), and VAE regularization. (A) DEN training schematic. Compared to **Supplementary Figure 19**, we use a VAE, pretrained as shown in **Supplementary Figure 20**, to estimate the marginal $p_{VAE}(x)$ of a generated sequence x, and we add a VAE component to the loss function set to $\max\left(0, \text{margin}_{VAE} - \log\left(p_{VAE}(x)\right)\right)$. Weights for the fitness, similarity, entropy, and VAE components of the loss function were 0.3, 5, 1, and 0.5. Margin values for the similarity, entropy, and VAE terms were 0.3, 1.8, and -30. Training was performed for 100 epochs. **(B)** Colormap representation of 50 5'UTR sequences randomly chosen from the 1,024 generated by the trained DEN. Each row represents a separate sequence, and color indicates nucleotide identity. **(C)** Distribution of edit distances per nucleotide, for 500 random pairs chosen from the 25nt random-end MPRA library (left) or the 1,024 sequences generated by the DEN. **(D)** Distribution of MRLs measured from the MPRA library (left), or predicted by Optimus 5-Prime(25) on all 1,024 DEN-generated sequences (middle) and the two selected sequences from this set (right). Source data are provided as a Source Data file.

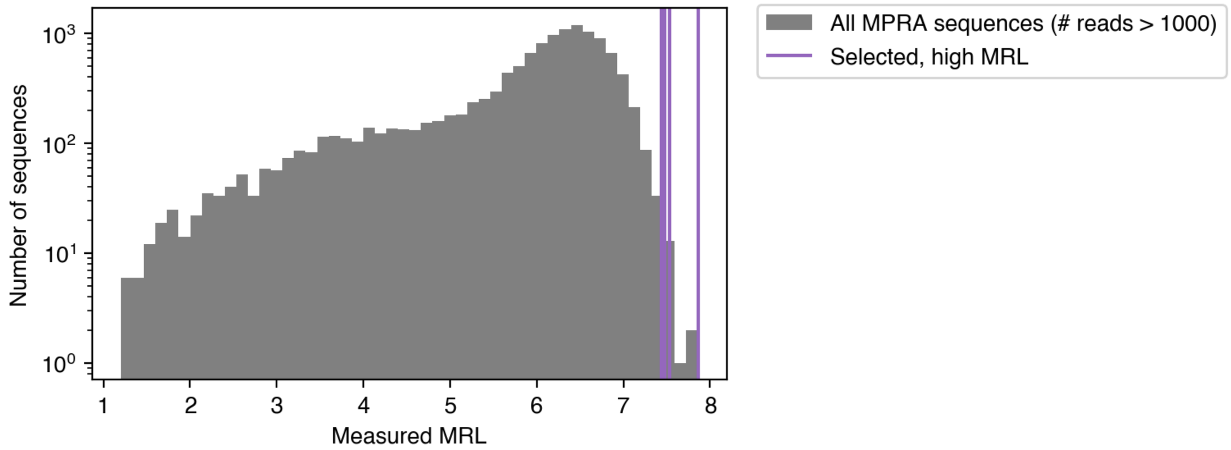

Supplementary Figure 22. Random-end 25nt MPRA high MRL controls for the megaTAL gene editing assays. (A) Starting from the random-end 25nt MPRA library data in HEK293T, we excluded sequences if their read count was lower than 1,000 or if they contained uATGs. The remaining sequences were sorted by MRL, and four from the top twenty were selected. The histogram shows the measured MRLs of all four control sequences, compared with a high-coverage (# reads > 1,000) subset of the MPRA library. Source data are provided as a Source Data file.

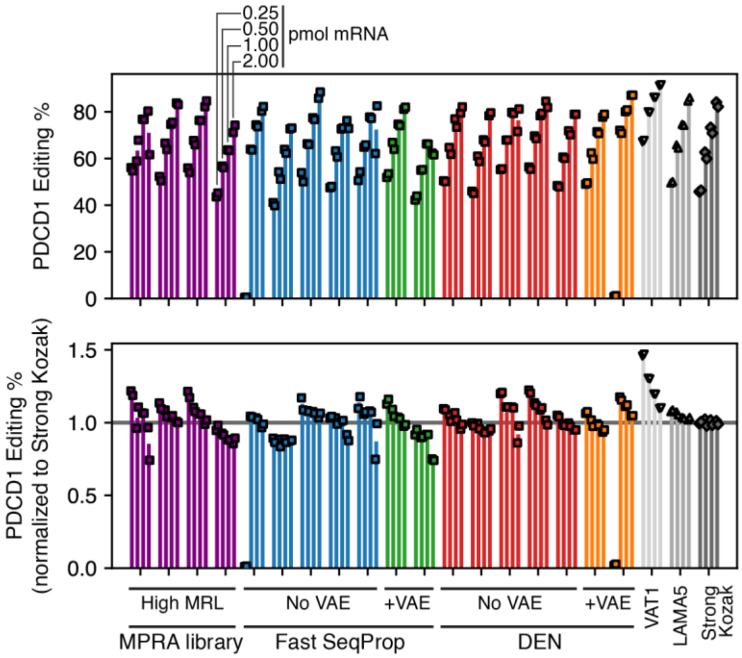

Supplementary Figure 23. Performance of the 25nt 5'UTR designs on the *PDCD1* megaTAL. Editing efficiencies for mRNAs with a megaTAL targeting the *PDCD1* gene, for 21 different 5'UTRs including designs and controls. Top: absolute editing efficiencies. Bottom: editing efficiencies normalized to the Strong Kozak control. Editing efficiencies for the first No VAE Fast SeqProp design and the second +VAE DEN design were close to zero only at a dosage of 0.25 pmol mRNA, and were deemed to be the result of experimental error and excluded from subsequent analysis. Analogous to **Figure 4A** and **B** but with the *PDCD1* megaTAL instead of *TGFBR2*. Source data are provided as a Source Data file.

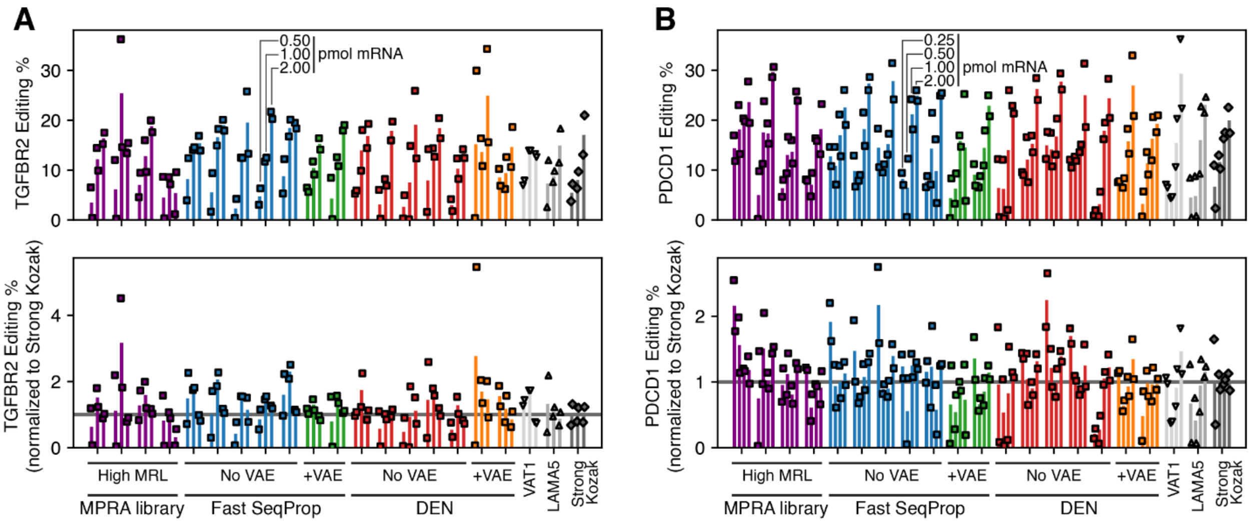

Supplementary Figure 24. Performance of 25nt 5'UTR designs on *TGFBR2* and *PDCD1* megaTALs in HepG2. Editing efficiencies for mRNAs with a megaTAL targeting the *TGFBR2* **(A)** or *PDCD1* **(B)** genes in HepG2 cells, for 21 different 5'UTRs including designs and controls. Top: absolute editing efficiencies. Bottom: editing efficiencies normalized to the Strong Kozak control. Analogous to **Figure 4A-B** and **Supplementary Figure 23** but using HepG2 instead of K562. Source data are provided as a Source Data file.

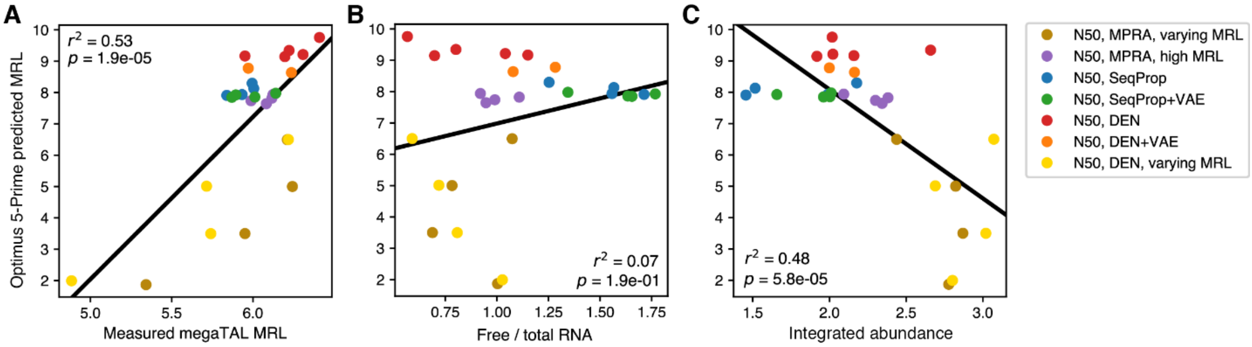

Supplementary Figure 25. Comparison of direct measurements of translation and stability of megaTAL mRNAs with Optimus 5-Prime predictions. Panels show predicted MRL compared to **(A)** measured MRL, **(B)** mRNA abundance in the ribosome-free fraction normalized to the total cell contents, and **(C)** integrated abundance over the abundance kinetics experiment. Solid lines are linear regression fits to all datapoints. p values from a two-sided Wald Test with t-distribution on the test statistic, performed on the linear regression slope with 0 as null hypothesis. No adjustment made for multiple comparisons. Source data are provided as a Source Data file.

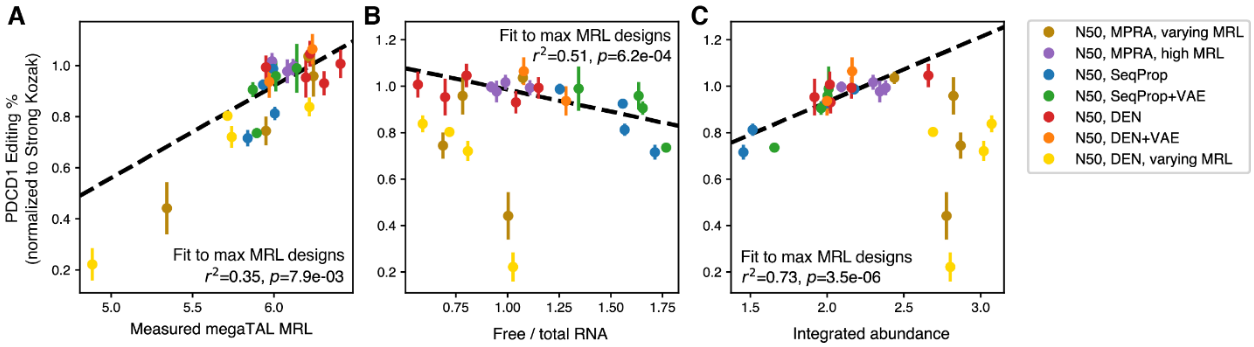

Supplementary Figure 26. Comparison of direct measurements of translation and stability of megaTAL mRNAs with *PDCD1* editing efficiency. Panels show editing efficiency compared to **(A)** measured MRL, **(B)** mRNA abundance in the ribosome-free fraction normalized to the total cell contents, and **(C)** integrated abundance over the abundance kinetics experiment. Panels are analogous to Figure 5B, E, and H, respectively. Each marker and error bar represent the mean and standard deviation of the Kozak-normalized editing efficiencies (Bottom panel of **Supplementary Figure 11A**) across all mRNA dosages for a particular 5'UTR (n=6 or 8: 3 or 4 dosages across two biological replicates per dosage). Dashed lines are linear regression fits to datapoints corresponding to 5'UTRs designed or selected for maximal MRL (no varying MRL sequences). p values from a two-sided Wald Test with t-distribution on the test statistic, performed on the linear regression slope with 0 as null hypothesis. No adjustment made for multiple comparisons. Source data are provided as a Source Data file.

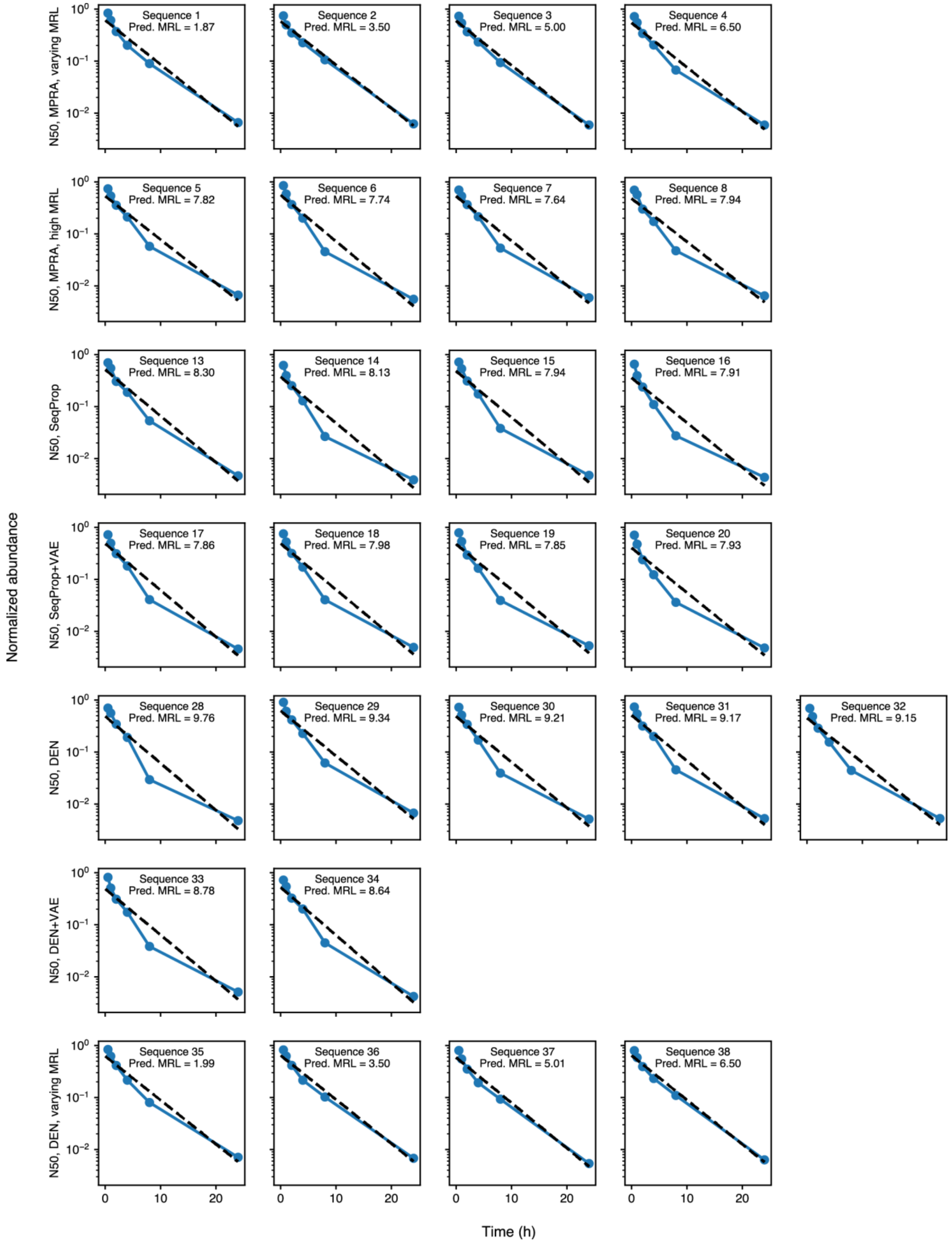

Normalized abundance
Time (h)
N50, MPRA, varying MRL
N50, MPRA, high MRL
N50, SeqProp
N50, SeqProp+VAE
N50, DEN
N50, DEN+VAE
N50, DEN, varying MRL
Sequence 1 Pred. MRL = 1.87
Sequence 2 Pred. MRL = 3.50
Sequence 3 Pred. MRL = 5.00
Sequence 4 Pred. MRL = 6.50
Sequence 5 Pred. MRL = 7.82
Sequence 6 Pred. MRL = 7.74
Sequence 7 Pred. MRL = 7.64
Sequence 8 Pred. MRL = 7.94
Sequence 13 Pred. MRL = 8.30
Sequence 14 Pred. MRL = 8.13
Sequence 15 Pred. MRL = 7.94
Sequence 16 Pred. MRL = 7.91
Sequence 17 Pred. MRL = 7.86
Sequence 18 Pred. MRL = 7.98
Sequence 19 Pred. MRL = 7.85
Sequence 20 Pred. MRL = 7.93
Sequence 28 Pred. MRL = 9.76
Sequence 29 Pred. MRL = 9.34
Sequence 30 Pred. MRL = 9.21
Sequence 31 Pred. MRL = 9.17
Sequence 32 Pred. MRL = 9.15
Sequence 33 Pred. MRL = 8.78
Sequence 34 Pred. MRL = 8.64
Sequence 35 Pred. MRL = 1.99
Sequence 36 Pred. MRL = 3.50
Sequence 37 Pred. MRL = 5.01
Sequence 38 Pred. MRL = 6.50

Supplementary Figure 27. megaTAL mRNA decay kinetics for all defined-end 50nt 5'UTRs. Blue lines correspond to measured abundances after normalization to spike-ins and initial IVT abundances (**Methods**). Dashed black lines are best fits to exponential decay curves of the form $y = \exp\left(-\frac{\mathrm{t}}{\tau} + \mathrm{b}\right)$. Source data are provided as a Source Data file.

References

1. Sample, P. J. *et al.* Human 5′ UTR design and variant effect prediction from a massively parallel translation assay. *Nat. Biotechnol.* **37**, 803–809 (2019).

2. Linder, J., Bogard, N., Rosenberg, A. B. & Seelig, G. A Generative Neural Network for Maximizing Fitness and Diversity of Synthetic DNA and Protein Sequences. *Cell Syst.* **11**, 49-62.e16 (2020).
