# Supplementary material for: Optimizing 5’UTRs for mRNA-delivered gene editing using deep learning
Source: Nat Commun. 2024 Jun 20;15:5284. doi: 10.1038/s41467-024-49508-2 (PMC11189900; doi:10.1038/s41467-024-49508-2)
Supplement: Supplementary file 3 — Description of Additional Supplementary Files [file 41467_2024_49508_MOESM3_ESM.pdf]

Description of Additional Supplementary Files

Supplementary Data 1. Processed megaTAL gene editing data

Supplementary Data 2. Processed megaTAL polysome profiling and stability UMI counts
