# Supplementary material for: Optimizing 5’UTRs for mRNA-delivered gene editing using deep learning
Source: Nat Commun. 2024 Jun 20;15:5284. doi: 10.1038/s41467-024-49508-2 (PMC11189900; doi:10.1038/s41467-024-49508-2)
Supplement: Supplementary file 6 — Reporting Summary [file 41467_2024_49508_MOESM6_ESM.pdf]

nature portfolio

Corresponding author(s): Georg Seelig

Last updated by author(s): May 31, 2024

Reporting Summary

Nature Portfolio wishes to improve the reproducibility of the work that we publish. This form provides structure for consistency and transparency in reporting. For further information on Nature Portfolio policies, see our Editorial Policies and the Editorial Policy Checklist.

Statistics

For all statistical analyses, confirm that the following items are present in the figure legend, table legend, main text, or Methods section.

n/a	Confirmed	
☐	☒	The exact sample size (*n*) for each experimental group/condition, given as a discrete number and unit of measurement
☐	☒	A statement on whether measurements were taken from distinct samples or whether the same sample was measured repeatedly
☐	☒	The statistical test(s) used AND whether they are one- or two-sided<br>*Only common tests should be described solely by name; describe more complex techniques in the Methods section.*
☒	☐	A description of all covariates tested
☐	☒	A description of any assumptions or corrections, such as tests of normality and adjustment for multiple comparisons
☐	☒	A full description of the statistical parameters including central tendency (e.g. means) or other basic estimates (e.g. regression coefficient) AND variation (e.g. standard deviation) or associated estimates of uncertainty (e.g. confidence intervals)
☐	☒	For null hypothesis testing, the test statistic (e.g. *F*, *t*, *r*) with confidence intervals, effect sizes, degrees of freedom and *P* value noted<br>*Give P values as exact values whenever suitable.*
☒	☐	For Bayesian analysis, information on the choice of priors and Markov chain Monte Carlo settings
☒	☐	For hierarchical and complex designs, identification of the appropriate level for tests and full reporting of outcomes
☒	☐	Estimates of effect sizes (e.g. Cohen's *d*, Pearson's *r*), indicating how they were calculated

Our web collection on statistics for biologists contains articles on many of the points above.

Software and code

Policy information about availability of computer code

Data collection	No custom code was used to collect the data in this study.
Data analysis	Code for analysis of polysome profiling results, deep learning modeling and sequence design, and gene editing analysis results has been deposited to github (https://github.com/castillohair/paper-5utr-design) and Zenodo (https://doi.org/10.5281/zenodo.11403014). For sequence design we used the following packages: Fast SeqProp 0.1 (https://github.com/johli/seqprop/), DEN 0.1 (https://github.com/johli/genesis/), keras 2.2, and tensorflow 1.15.

For manuscripts utilizing custom algorithms or software that are central to the research but not yet described in published literature, software must be made available to editors and reviewers. We strongly encourage code deposition in a community repository (e.g. GitHub). See the Nature Portfolio guidelines for submitting code & software for further information.

Data

Policy information about availability of data

All manuscripts must include a data availability statement. This statement should provide the following information, where applicable:

- Accession codes, unique identifiers, or web links for publicly available datasets
- A description of any restrictions on data availability
- For clinical datasets or third party data, please ensure that the statement adheres to our policy

The polysome profiling data generated in this study (raw fastq files and processed UMI counts) has been deposited in the Gene Expression Omnibus under accession code GSE232927 [https://www.ncbi.nlm.nih.gov/geo/query/acc.cgi?acc=GSE232927]. The polysome profiling data from the fixed-end N50 library from our previous

publication can be found in the Gene Expression Omnibus under accession code GSE114002 [https://www.ncbi.nlm.nih.gov/geo/query/acc.cgi?acc=GSE114002]. The processed megaTAL gene editing data is provided in Supplementary Data 1. The processed megaTAL polysome profiling and stability UMI counts are provided in Supplementary Data 2. Source data for every figure in this paper has been deposited to Zenodo [https://doi.org/10.5281/zenodo.11398662].

Research involving human participants, their data, or biological material

Policy information about studies with human participants or human data. See also policy information about sex, gender (identity/presentation), and sexual orientation and race, ethnicity and racism.

Reporting on sex and gender	N/A
Reporting on race, ethnicity, or other socially relevant groupings	N/A
Population characteristics	N/A
Recruitment	N/A
Ethics oversight	N/A

Note that full information on the approval of the study protocol must also be provided in the manuscript.

Field-specific reporting

Please select the one below that is the best fit for your research. If you are not sure, read the appropriate sections before making your selection.

☒ Life sciences ☐ Behavioural & social sciences ☐ Ecological, evolutionary & environmental sciences

For a reference copy of the document with all sections, see nature.com/documents/nr-reporting-summary-flat.pdf

Life sciences study design

All studies must disclose on these points even when the disclosure is negative.

Sample size	Sample size was not statistically predetermined. in vitro experiments were performed in biological replicate according to general practice in the field.
Data exclusions	In polysome profiling experiments, 5'UTR sequences with fewer than 100 reads across all polysome fractions were excluded, according to general practice and our previous publication.
Replication	Polysome profiling experiments were performed as two biological replicates in T cells and one in HepG2, and two for the N25 random-end library and one for the N50 library in HEK293T cells. Gene editing experiments were performed in duplicate for each 5'UTR design, cell line, target gene, and mRNA dosage. The results were confirmed to be consistent at four different mRNA dosages for each 5'UTR design, cell line, and target gene.
Randomization	No randomization was performed since the results are in vitro assays.
Blinding	No blinding was performed since the results are quantitative measurements from instruments on non-patient samples.

Reporting for specific materials, systems and methods

We require information from authors about some types of materials, experimental systems and methods used in many studies. Here, indicate whether each material, system or method listed is relevant to your study. If you are not sure if a list item applies to your research, read the appropriate section before selecting a response.

Materials & experimental systems

n/a	Involved in the study	
☒	☐	Antibodies
☐	☒	Eukaryotic cell lines
☒	☐	Palaeontology and archaeology
☒	☐	Animals and other organisms
☒	☐	Clinical data
☒	☐	Dual use research of concern
☒	☐	Plants

Methods

n/a	Involved in the study	
☒	☐	ChIP-seq
☒	☐	Flow cytometry
☒	☐	MRI-based neuroimaging

Eukaryotic cell lines

Policy information about cell lines and Sex and Gender in Research

Cell line source(s)	HEK293T (CRL-3216), K562 (CCL-243), and HepG2 (HB-8065) cells were sourced from ATCC. PBMCs for T cell work were purchased from Key Biologics (currently Charles River).
Authentication	Cell lines were authenticated by STR profiling at ATCC.
Mycoplasma contamination	Cell lines were not tested for mycoplasma during the course of this study but were confirmed negative for mycoplasma by ATCC.
Commonly misidentified lines (See ICLAC register)	No commonly misidentified cell lines were used in this study.
